# Have Almost Fifty Years Of Disability Civil Rights Laws Achieved Equitable Care?

**DOI:** 10.1377/hlthaff.2022.00413

**Published:** 2022-10

**Authors:** Lisa I. Iezzoni, Michael M. McKee, Michelle A. Meade, Megan A. Morris, Elizabeth Pendo

**Affiliations:** Harvard University and Massachusetts General Hospital, Boston, Massachusetts.; University of Michigan, Ann Arbor, Michigan.; University of Michigan, Ann Arbor.; University of Colorado, Aurora, Colorado.; Saint Louis University, St. Louis, Missouri.

## Abstract

For almost fifty years, federal civil rights laws such as Section 504 of the Rehabilitation Act of 1973, the Americans with Disabilities Act (ADA) of 1990 and the ADA Amendments Act of 2008, and Section 1557 and other provisions of the 2010 Patient Protection and Affordable Care Act have prohibited discrimination against Americans with disabilities, including in health care. Despite these laws, disabled Americans continue to experience disparities in health and health care, from preventive care to home and community-based services. In its 2022 *Health Equity Framework for People with Disabilities*, the National Council on Disability highlighted some of these disparities and recommended remedies. To explore these concerns, this article examines disability inequities and potential solutions within six areas. It concludes by recommending the ratification of the 2006 United Nations Convention on the Rights of Persons with Disabilities to reinvigorate US efforts to maximize the health and dignity of disabled Americans and support their full participation in the community.

In its February 2022 *Health Equity Framework for People with Disabilities*, the National Council on Disability (NCD) called for “an all-of-government approach to achieve health equity…for the largest unrecognized minority group in this country, the over 61 million people with disabilities.”^1^ The NCD summarizes diverse and persistent disparities in health and health care affecting Americans with disabilities, noting that systemic barriers within US health care cause these shortfalls. Advancing equity for people with disabilities, it asserts, therefore requires fundamental changes and ongoing societal commitments across all public and private health care sectors.^1^

The enduring health and health care disparities disadvantaging Americans with disabilities^2^ are discouraging, given the nearly half-century of civil rights laws intended to achieve equity for disabled people. Section 504 of the Rehabilitation Act of 1973 requires that programs receiving federal funds, including Medicare and Medicaid, ensure equitable access for disabled Americans. The Americans with Disabilities Act (ADA) of 1990 and the ADA Amendments Act of 2008, which clarified definitions of *disability*, extended these civil rights protections to other public and private settings and services. Section 1557 of the 2010 Patient Protection and Affordable Care Act (ACA) amended Section 504 of the Rehabilitation Act and several other statutes to provide additional protections against disability discrimination in health care services. Nevertheless, disabled Americans experience disparities and inadequate services across the health care continuum, from preventive care to home and community-based services.^1,2^

This article highlights six areas with persistent health and health care inequities for disabled Americans and suggests potential remedies: disability data gaps, accessible communication, physical access to care, competency training across health professions, home and community-based supports, and nondiscriminatory health insurance benefit design. Some of these issues have obvious solutions, whereas others are more intractable. We conclude by recommending that US policy makers revisit and consider ratifying the 2006 United Nations Convention on the Rights of Persons with Disabilities.^3,4^ Recognizing differing preferences, this article alternates between person-first and identity-first disability language.^5^

## Disability Data Gaps

Public health and health care delivery systems do not routinely gather disability information.^6^ Diagnosis codes—the primary clinical information in administrative files—provide little insight into disability. Most population-based information about health and health care disparities for disabled Americans therefore comes from surveys. Almost thirty years ago, shortly after the ADA’s enactment, the National Health Interview Survey Disability Supplement (NHIS-D, 1994–95) was conducted under a unique partnership across four federal agencies.^7^ The NHIS-D gathered not only health-related information but also data about transportation, housing, and other sectors that gave insights into social determinants of health. Subsequent federal and state surveys have collected disability information, but without the NHIS-D’s broad scope. Inconsistent disability questions across surveys have impeded the interpretation and comparison of results.^8^

To facilitate disparities analyses and improve data quality, Section 4302 of the ACA required the Department of Health and Human Services to develop standards for federal data collection and reporting on five demographic attributes: ethnicity, race, sex, primary language, and disability status. The six disability questions from the Census Bureau’s American Community Survey (ACS-6) were selected as the standard. Despite reservations about the ACS-6 (such as whether the six questions identify certain subgroups of disabled people),^9^ many federal surveys now gather data on disability status using these questions.

As demonstrated during the COVID-19 pandemic, the absence of disability data in public health and health care delivery systems impedes efforts to monitor and manage the health care experiences of people with disabilities.^10^ Furthermore, this “lack of data perpetuates the exclusion of disabled people from discussions of health equity and policies that are data driven.”^10^ Electronic health records also do not consistently capture disability status, despite the need for this information to support clinical care and facilitate the timely provision of reasonable accommodations during health care encounters.^11^ In its 2022 *Health Equity Framework*, the NCD included improving disability data collection across the life span among its top priorities.^1^

## Accessible Communication

Effective communication between patients and clinicians is essential to ensuring high-quality care and is required under the ADA and Rehabilitation Act Section 504. For people with disabilities that affect oral or written communication (for example, relating to hearing, vision, and speech), various auxiliary aids, telecommunication methods, and other services facilitate effective communication. However, research suggests that patients often do not receive these accommodations. For example, deaf patients report that some physicians communicate through note writing, lip reading, talking slowly, or shouting rather than providing sign language interpreters or other accommodations.^12,13^ For patients with hearing loss, communication breakdowns with clinicians can reduce treatment adherence and cause worse health outcomes.^14^ People with vision impairments also experience health and health care inequities, perpetuated by inaccessible communication with clinicians.^15^ A 2019–20 US national survey found that more than half of outpatient physicians never (36.7 percent) or rarely (19.0 percent) provide printed materials in large type, and more than half never (23.8 percent) or rarely (26.4 percent) give a spoken description of the exam room to their patients with limited vision,^16^ even though patients with vision impairments have indicated that these strategies would improve their health care experiences.^17^

Physicians sometimes recognize that they fail to accommodate disabled patients’ communication preferences, blaming logistical concerns, costs, or ignorance about communication options.^13,18^ Not surprisingly, a large fraction of ADA lawsuits involve failures to ensure effective communication.^19^ In the 2019–20 survey, 35.8 percent of physicians reported knowing little or nothing about their legal responsibilities under the ADA, and 71.2 percent responded incorrectly about who determines reasonable accommodations for patients with disabilities (these decisions require collaboration between patients and clinicians).^20^ To make communication accessible, clinicians need more training about their legal obligations and on strategies and accommodations to address communication barriers. The ADA recognizes that “there is no one-size-fits-all solution [for the] provision of auxiliary aids and services.”^19^ Asking patients which communication accommodations work best for them and then following their preferences would maximize communication access and reduce lawsuit risks.^13,19^

## Physical Access To Care

Although the ADA requires that disabled Americans receive equitable access to health care, detailed ADA regulations on physical spaces apply only to fixed structures, not medical equipment and furnishings such as examination tables and diagnostic imaging equipment. Over time, especially in nonrural settings, health care facilities have eliminated most structural physical barriers with renovations to install ramps, enlarge bathrooms, affix grab bars, and make other modifications. An accessibility survey administered from 2006 to 2010 to 2,389 provider sites contracting with California Medicaid managed care plans^21^ found that van-accessible parking was inadequate, but other parking spaces, exterior access, building access, and interior public spaces generally complied with structural access requirements. However, only 8.4 percent of sites had a height-adjustable (that is, accessible) exam table, and just 3.6 percent had a roll-on weight scale.^21^

Inaccessible medical diagnostic equipment contributes to disparities and poor-quality care for people with mobility disabilities. Without such equipment, patients may be examined in their wheelchairs when complete physical examinations are clinically indicated, and they might not receive routine services such as weight measurement and Pap tests.^22-24^ Recognizing these and other adverse consequences, some congressional leaders tried throughout the mid-2000s to pass legislation requiring specification of medical diagnostic equipment accessibility standards, but these efforts repeatedly stalled.

Section 4203 of the ACA required the US Access Board, in collaboration with the Food and Drug Administration, to develop standards that “shall ensure that such equipment…allow independent entry to, use of, and exit from the equipment…to the extent possible.”^25^ Over the course of several years, these entities developed accessibility standards for exam tables and chairs, weight scales, diagnostic imaging equipment, mammography machines, and gurneys and stretchers, and they shepherded those standards through the federal rulemaking process; the final rule appeared January 9, 2017.^26^ The next step was for the Department of Justice to adopt the standards as ADA regulations and specify requirements for accessible equipment availability. However, on December 26, 2017, the Trump administration suspended this rulemaking process.^27^

Although no mandates currently require health care settings to have medical diagnostic equipment meeting the approved accessibility standards, many health care facilities have acquired accessible equipment in recent years. Height-adjustable exam tables can improve patients’ sense of safety^28^ and reduce occupational injury risks to staff.^29^ Despite these and other benefits, however, most US physicians practicing in outpatient settings do not use accessible equipment. In 2019–20 only 22.6 percent of US outpatient physicians reported always or usually using accessible weight scales for patients with significant mobility limitations, and 40.3 percent always or usually used accessible exam tables or chairs.^30^

Requiring accessible medical diagnostic equipment is another top priority of the NCD’s 2022 *Health Equity Framework*.^1^ The NCD recommends adoption of the approved equipment accessibility standards by the Department of Justice and the Office for Civil Rights in the Department of Health and Human Services, which lists preventing discrimination through provision of accessible medical equipment as a fiscal year 2022 regulatory priority.^31^

## Disability Competency Training Across Health Care Professions

Discriminatory or ableist attitudes persist throughout the health care delivery system and contribute to ongoing health care disparities.^1^ In one study examining results from implicit association tests, although health care professionals across disciplines self-reported little explicit prejudice against disabled people, their implicit attitudes revealed ableist bias.^32^ In the 2019–20 national survey, 82.4 percent of outpatient physicians reported that people with significant disability have worse quality of life than nondisabled people, and only 40.7 percent were very confident that they could provide the same quality of care to patients with disability as to their nondisabled patients.^33^ Erroneous assumptions about disabled people affect their quality of care. For instance, clinicians may believe that people with hearing loss have low intelligence and disrespect their perspectives.^13^ Also, physicians often erroneously assume that disabled people are not sexually active and thus are not at risk for human papillomavirus exposure; they therefore fail to recommend cervical cancer screening.^34^

Relatively few undergraduate or graduate medical education programs offer disability training.^35,36^ Evaluations of these disability education initiatives generally have found immediate beneficial changes in attitudes, skills, and knowledge among trainees; little research has assessed long-term effects, including whether these efforts improve patient care.^35^ Increasing the number of disabled people in medical school could catalyze or bolster disability competence among physicians.^37^ However, considerable barriers impede people with disabilities from pursuing training at US medical schools,^38^ and only 3.1 percent of practicing physicians in a 2019 US national survey self-identified as having disability.^39^

ACA Section 5307 authorizes federal funding to train health care professionals in disability competent care and requires an assessment of how many health care organizations’ employees have received this training. Despite this requirement, no such assessment has been conducted. Nevertheless, components of disability competency for health care professionals have been conceptualized, and efforts to develop disability competency training programs involving interprofessional collaborations are underway.^40,41^ Through the Alliance for Disability in Health Care Education, representatives from medicine, nursing, psychology, physical therapy, occupational therapy, and other allied health professions have worked since 2012 to reach consensus about disability curriculum standards for training health care professionals.^41^ Through two waves of consensus-building efforts involving people with disabilities, their family members, and disability advocates, the alliance delineated six competencies, forty-nine subcompetencies, and ten principles and values, including respect and awareness of communication and physical barriers. Adopting competency-based disability training will require time and cooperative efforts by a wide range of stakeholders, including professional associations, accreditation bodies, and individual educators.^41^ Implementing comprehensive disability competency training for health care professionals is another top priority of the NCD’s 2022 *Health Equity Framework*.^1^

## Home And Community-Based Supports

People with disabilities who require in-home assistance with activities of daily living generally receive support from family members or friends, whereas others need paid personal assistance services to supplement informal caregivers or because they lack family or friends to provide in-home support.^42^ The ADA envisions full lives for disabled people regardless of their support needs. ADA Section 12101(a)(1) states, “Physical or mental disabilities in no way diminish a person’s right to fully participate in all aspects of society,” and Section 12101(a)(7) asserts the goal of assuring “equality of opportunity, full participation, independent living, and economic self-sufficiency” for people with disabilities.

The late Justice Ruth Bader Ginsberg partially relied on these concepts in writing the Supreme Court’s 1999 landmark decision in *Olmstead v. L. C.* This ruling held that under the ADA Title II, states cannot confine people with disabilities to institutions—they have the civil right to live within communities with appropriate supports, in this case funded by public insurance, Medicaid.^43^ However, in *Olmstead*, disability civil rights and Medicaid payment policies collided: “The Court had to wrestle with the extent to which the ADA requires state Medicaid programs to make deep and structural reforms in how long-term care services are organized, financed, and delivered.”^44^ The Supreme Court does not have the authority to require state Medicaid programs to cover home and community-based services, so it suggested that putting Medicaid beneficiaries on waiting lists for these services would be acceptable if this would not generate lengthy delays.

To advance equity in the context of home and community-based services for disabled people, it is important to understand two critical points. First, although costs vary depending on a person’s support needs, home and community-based services are too expensive for most Americans. Fewpeople have private insurance to cover these costs. For years, seemingly intractable actuarial, cost, political, and other problems have sunk efforts to develop public insurance for long-term support services for Americans with Disabilities, such as the CLASS Act (Community Living Assistance Services and Supports, ACA Title VIII, which was repealed in 2013).^45^ Medicaid has become “a last resort” for thousands of people who cannot afford home and community-based services.^46^ However, Medicaid eligibility criteria, which differ considerably across states, often make it difficult or impossible for many who need services to qualify for benefits. In the mid-1970s personal care became an optional Medicaid benefit. The Omnibus Budget Reconciliation Act of 1981 introduced Section 1915(c) waivers, permitting Medicaid to cover home and community-based services, including personal care, homemaker and home health aide services, and respite for family caregivers.^47^ This authority and new Medicaid waiver options, including some mandated in the ACA, have galvanized many states to move beneficiaries with Disabilities from institutions into communities. In fiscal year 2013, for the first time, the proportion of Medicaid long-term services and supports funds spent on home and community-based services (51 percent) exceeded the proportion used for institutional care (49 percent).^48^

Second, Medicaid home and community-based services programs vary substantially across states “in enrollment and spending, reflecting states’ different choices about optional authorities, benefit package contents, and scope of covered services.”^49^ For example, in 2016 Mississippi spent 27 percent of its Medicaid long-term services and supports funding on home and community-based services, whereas Oregon allocated 81 percent of its Medicaid long-term services and supports dollars to these services.^50^ Nevertheless, home and community-based services funding remains constrained even in states prioritizing these services, resulting in long waiting lists that also vary among states, from less than one year up to fourteen years.^51^ In 2018 Medicaid home and community-based services waiver waiting lists contained nearly 820,000 people nationally, with average wait times of thirty-nine months.^52^ The *Olmstead* decision did not indicate what would constitute lengthy waiting periods for Medicaid home and community-based services. To decrease barriers to these services for disabled Americans, the NCD recommends “vigorous enforcement of the [ADA] integration mandate and the *Olmstead* decision.”^1^ Conducting enforcement activities to increase access to home and community-based services for people with disabilities would require administrative action from the Department of Justice.^1^

## Nondiscriminatory Private Health Insurance Benefit Design

In 2020, 45.5 percent of insured Americans ages 15–64 with disabilities had private health insurance through employer-sponsored coverage or direct purchase in the individual market (in contrast, 74.4 percent of nondisabled people ages 15–64 had private insurance).^53^ Before the ACA’s enactment, complex, fragmented, and often restrictive private health insurance policies left many disabled people without coverage; others faced exclusions, limits, cost-sharing obligations, and high costs that prevented them from obtaining needed care, equipment, and services.^2^ The ADA’s “safe harbor” exception allowed some health insurance plans to exclude or restrict coverage for people with preexisting health conditions, including disability.^54^

ACA reforms to insurance underwriting rules, affordability, and benefit design in the individual and group insurance markets addressed some of these problems. Section 1557, the ACA’s non-discrimination provision, was initially viewed as an important new way to forestall benefit exclusions or denials based on disability. In the Obama administration, extensive rulemaking to implement Section 1557 prevented health insurers and health care delivery systems from discriminating on the basis of age, sex, race, national origin, or disability by denying, canceling, limiting, or refusing to issue or renew a health insurance policy; denying or limiting coverage of health insurance claims; imposing additional cost sharing or other limitations or restrictions on coverage; or using discriminatory marketing practices or insurance benefit designs.^55^

Instances of potentially discriminatory benefit design were intended to be analyzed case by case. However, the Trump administration promulgated rules that substantially narrowed the scope of Section 1557 and eliminated provisions prohibiting discrimination in plan benefit design.^56^ To achieve equity in access to health coverage for people with disabilities, revising Section 1557 to reinstate its broad protections is essential.^31,56,57^

In May 2022 the Biden administration issued a final rule for qualified health plans under the ACA that requires nondiscriminatory benefit design that “is clinically based”; any limitation on covered benefits based on “an individual's age, expected length of life, present or predicted disability, degree of medical dependency, quality of life, other health conditions, race, color, national origin, disability, age, or sex, must be based on clinical evidence.”^58^ The rule identifies instances of presumptively discriminatory benefit design, such as applying age limitations on hearing aids or autism spectrum disorder coverage, limiting foot care coverage based on diagnosis (for example, diabetes), and placing all or a majority of drugs to treat certain conditions (for example, HIV and AIDS) in a high cost-sharing tier.^58^ The utility of Section 1557 for challenging discriminatory benefit design continues to evolve.

## Discussion

For nearly fifty years disability civil rights laws have failed to achieve equitable health and health care for Americans with disabilities. Solutions exist to address many inequities. However, remedies have not been widely or reliably implemented, thus perpetuating substandard care and worse health outcomes for people with Disabilities. It is therefore critical to reinvigorate societal commitments to upholding the dignity and rights of disabled people—a minority group that anyone can join in a flash.

The Convention on the Rights of Persons with Disabilities,^3^ approved by the UN General Assembly in December 2006 and ratified by 185 countries as of May 2022,^3^ offers a useful framework for realizing this goal.^4,15^ Signed but never ratified by the US,^4,59^ the convention contains fifty articles that address fundamental rights across societal sectors (such as rights to accessible health care, education, housing, and employment) to support active lives with dignity for people with disabilities. As Arlene Kanter wrote in 2015, “Whereas the ADA is a civil rights law that views equality for people with disabilities through a limited antidiscrimination lens, the [UN convention] is a human rights law that, based on a social model of disability, moves beyond traditional notions of equality towards a society that accepts people with disabilities as full and equal members.”^4^

The UN convention’s scope is broad and aspirational, and no evidence exists that any country has achieved its expansive goals. Nevertheless, as the number of Americans with disabilities grows, revisiting strategies to support their full participation in the community—and thus likely their health—is overdue and necessary. Ratifying the Convention on the Rights of Persons with Disabilities could energize efforts to improve the health, daily lives, and dignity of Americans with disabilities.

## References

[1] National Council on Disability. Health equity framework for people with disabilities [Internet]. Washington (DC): NCD; 2022 Feb [cited 2022 Jul 22]. Available from: https://www.ncd.gov/sites/default/files/NCD_Health_Equity_Framework.pdf

[2] National Council on Disability. The current state of health care for people with disabilities [Internet]. Washington (DC): NCD; 2009 Sep 30 [cited 2022 Jul 22]. Available from: https://files.eric.ed.gov/fulltext/ED507726.pdf

[3] United Nations Department of Economic and Social Affairs. Convention on the Rights of Persons with Disabilities (CRPD) [Internet]. New York (NY): UN; [cited 2022 Jul 22]. Available from: https://www.un.org/development/desa/disabilities/convention-on-the-rights-of-persons-with-disabilities.html

[4] Kanter AS. The Americans with Disabilities Act at 25 years: lessons to learn from the Convention on the Rights of People with Disabilities. Drake Law Rev. 2015;63:819–83.

[5] Andrews EE, Forber-Pratt AJ, Mona LR, Lund EM, Pilarski CR, Balter R. #SaytheWord: a disability culture commentary on the erasure of “disability”. Rehabil Psychol. 2019; 64(2):111–8. 30762412 10.1037/rep0000258

[6] Rios D, Magasi S, Novak C, Harniss M. Conducting accessible research: including people with disabilities in public health, epidemiological, and outcomes studies. Am J Public Health. 2016;106(12):2137–44. 27736212 10.2105/AJPH.2016.303448 PMC5104996

[7] Ward BW, Ridolfo H, Creamer L, Gray C. The 1994–1995 National Health Interview Survey on disability (NHIS-D): a bibliography of 20 years of research. Rev Disabil Stud. 2015;11(2):1–22. 26640424 PMC4666019

[8] Freedman VA, Crimmins E, Schoeni RF, Spillman BC, Aykan H, Kramarow E, Resolving inconsistencies in trends in old-age disability: report from a technical working group. Demography. 2004;41(3):417–41. 15461008 10.1353/dem.2004.0022

[9] Livermore G, Whalen D, Stapleton DC. Assessing the need for a national disability survey: final report [Internet]. Washington (DC): Department of Health and Human Services, Office of the Assistant Secretary for Planning and Evaluation; 2011 Sep [cited 2022 Jul 22]. Available from: https://aspe.hhs.gov/sites/default/files/migrated_legacy_files//43686/NatlDS.pdf

[10] Reed NS, Meeks LM, Swenor BK. Disability and COVID-19: who counts depends on who is counted. Lancet Public Health. 2020;5(8):e423. 32707124 10.1016/S2468-2667(20)30161-4 PMC7373402

[11] Morris MA, Hasnain-Wynia R. A research agenda for documenting disability status within healthcare organizations to address disparities in care. J Healthc Qual. 2014;36(2):7–12, quiz 12–3. 24350672 10.1111/jhq.12059

[12] James TG, Coady KA, Stacciarini JR, McKee MM, Phillips DG, Maruca D, “They’re not willing to accommodate Deaf patients”: communication experiences of Deaf American Sign Language users in the emergency department. Qual Health Res. 2022;32(1):48–63. 34823402 10.1177/10497323211046238

[13] Iezzoni LI, O’Day BL, Killeen M, Harker H. Communicating about health care: observations from persons who are deaf or hard of hearing. Ann Intern Med. 2004;140(5):356–62. 14996677 10.7326/0003-4819-140-5-200403020-00011

[14] McKee MM, Moreland C, Atcherson SR, Zazove P. Hearing loss: communicating with the patient who is deaf or hard of hearing. FP Essent. 2015;434:24–8. 26161525

[15] Ramke J, Ah Tong BAM, Swenor BK. Improving health care accessibility for people with vision impairment—a call to account. JAMA Ophthalmol. 2022;140(1):84–5. 34854896 10.1001/jamaophthalmol.2021.5069

[16] Iezzoni LI, Rao SR, Ressalam J, Bolcic-Jankovic D, Campbell EG. Incidence of accommodations for patients with significant vision limitations in physicians’ offices in the US. JAMA Ophthalmol. 2022;140(1):79–84. 34854912 10.1001/jamaophthalmol.2021.5072 PMC8640945

[17] Cupples ME, Hart PM, Johnston A, Jackson AJ. Improving healthcare access for people with visual impairment and blindness. BMJ. 2012;344:e542. 22290101 10.1136/bmj.e542

[18] Agaronnik N, Campbell EG, Ressalam J, Iezzoni LI. Communicating with patients with disability: perspectives of practicing physicians. J Gen Intern Med. 2019;34(7):1139–45. 30887435 10.1007/s11606-019-04911-0 PMC6614249

[19] Great Lakes ADA Center. The ADA and the healthcare industry [Internet]. Chicago (IL): The Center; 2019 May [cited 2022 Jul 22]. (Brief No. 42). Available from: https://www.adagreatlakes.org/Publications/Legal_Briefs/Brief_42_ADA_and_the_Healthcare_Industry.pdf

[20] Iezzoni LI, Rao SR, Ressalam J, Bolcic-Jankovic D, Agaronnik ND, Lagu T, US physicians’ knowledge about the Americans with Disabilities Act and accommodation of patients with disability. Health Aff (Millwood). 2022;41(1):96–104. 34982624 10.1377/hlthaff.2021.01136 PMC8740697

[21] Mudrick NR, Breslin ML, Liang M, Yee S. Physical accessibility in primary health care settings: results from California on-site reviews. Disabil Health J. 2012;5(3):159–67. 22726856 10.1016/j.dhjo.2012.02.002

[22] Pharr JR, James T, Yeung Y-L. Accessibility and accommodations for patients with mobility disabilities in a large healthcare system: how are we doing? Disabil Health J. 2019;12(4):679–84. 30940437 10.1016/j.dhjo.2019.03.008

[23] Frost KL, Bertocci G, Stillman MD, Smalley C, Williams S. Accessibility of outpatient healthcare providers for wheelchair users: pilot study. J Rehabil Res Dev. 2015;52(6):653–62. 26560684 10.1682/JRRD.2015.01.0002

[24] Morris MA, Maragh-Bass AC, Griffin JM, Finney Rutten LJ, Lagu T, Phelan S. Use of accessible examination tables in the primary care setting: a survey of physical evaluations and patient attitudes. J Gen Intern Med. 2017;32(12):1342–8. 28924919 10.1007/s11606-017-4155-2 PMC5698222

[25] Iezzoni LI, Pendo E. Accessibility of medical diagnostic equipment—implications for people with disability. N Engl J Med. 2018;378(15):1371–3. 29641961 10.1056/NEJMp1800606

[26] Architectural and Transportation Barriers Compliance Board. Standards for accessible medical diagnostic equipment. Final rule. Fed Regist. 2017;82(5):2810–48. 28071879

[27] Department of Justice. Non-discrimination on the basis of disability; notice of withdrawal of four previously announced rulemaking actions. Fed Regist. 2017;82(246):60932–3.

[28] Fragala G, Labreche M, Wawzynieki P. Benefits achieved for patients through application of height-adjustable examination tables. J Patient Exp. 2017;4(3):138–43. 28959719 10.1177/2374373517706835 PMC5593265

[29] Fragala G. Reducing occupational risk to ambulatory caregivers. Workplace Health Saf. 2016;64(9):414–9. 27174130 10.1177/2165079916642776

[30] Iezzoni LI, Rao SR, Ressalam J, Bolcic-Jankovic D, Donelan K, Agaronnik N, Use of accessible weight scales and examination tables/chairs for patients with significant mobility limitations by physicians nationwide. Jt Comm J Qual Patient Saf. 2021;47(10):615–26. 34364797 10.1016/j.jcjq.2021.06.005 PMC8464497

[31] Department of Health and Human Services. Statement of regulatory priorities for fiscal year 2022 [Internet]. Washington (DC): HHS; 2021 [cited 2022 Jul 22]. Available from: https://www.reginfo.gov/public/jsp/eAgenda/StaticContent/202110/Statement_0900_HHS.pdf

[32] VanPuymbrouck L, Friedman C, Feldner H. Explicit and implicit disability attitudes of healthcare providers. Rehabil Psychol. 2020;65(2):101–12. 32105109 10.1037/rep0000317 PMC9534792

[33] Iezzoni LI, Rao SR, Ressalam J, Bolcic-Jankovic D, Agaronnik ND, Donelan K, Physicians’ perceptions of people with disability and their health care. Health Aff (Millwood). 2021;40(2):297–306. 33523739 10.1377/hlthaff.2020.01452 PMC8722582

[34] Chen LS, Chou YJ, Tsay JH, Lee CH, Chou P, Huang N. Variation in the cervical cancer screening compliance among women with disability. J Med Screen. 2009;16(2):85–90. 19564521 10.1258/jms.2009.008061

[35] Ioerger M, Flanders RM, French-Lawyer JR, Turk MA. Interventions to teach medical students about disability: a systematic search and review. Am J Phys Med Rehabil. 2019;98(7):577–99. 30730327 10.1097/PHM.0000000000001154

[36] Sapp RW, Sebok-Syer SS, Gisondi MA, Rotoli JM, Backster A, McClure Poffenberger C. The prevalence of disability health training and residents with disabilities in emergency medicine residency programs. AEM Educ Train. 2020;5(2):e10511. 33898914 10.1002/aet2.10511 PMC8053000

[37] Iezzoni LI. Why increasing numbers of physicians with disability could improve care for patients with disability. AMA J Ethics. 2016;18(10):1041–9. 27780029 10.1001/journalofethics.2016.18.10.msoc2-1610

[38] Curry RH, Meeks LM, Iezzoni LI. Beyond technical standards: a competency-based framework for access and inclusion in medical education. Acad Med. 2020;95(12S Addressing Harmful Bias and Eliminating Discrimination in Health Professions Learning Environments, 12s):S109–12. 10.1097/ACM.0000000000003686 32889921

[39] Nouri Z, Dill MJ, Conrad SS, Moreland CJ, Meeks LM. Estimated prevalence of US physicians with disabilities. JAMA Netw Open. 2021;4(3):e211254. 33710286 10.1001/jamanetworkopen.2021.1254 PMC7955270

[40] Ankam NS, Bosques G, Sauter C, Stiens S, Therattil M, Williams FH, Competency-based curriculum development to meet the needs of people with disabilities: a call to action. Acad Med. 2019;94(6):781–8. 30844926 10.1097/ACM.0000000000002686

[41] Havercamp SM, Barnhart WR, Robinson AC, Whalen Smith CN. What should we teach about disability? National consensus on disability competencies for health care education. Disabil Health J. 2021;14(2):100989. 32952097 10.1016/j.dhjo.2020.100989

[42] Iezzoni LI. Making their days happen. Paid personal assistance services for people with disability living in their home and communities. Philadelphia (PA): Temple University Press; 2022.

[43] Rosenbaum S. The *Olmstead* decision: implications for state health policy. Health Aff (Millwood). 2000;19(5):228–32. 10992673 10.1377/hlthaff.19.5.228

[44] Rosenbaum S. Using the courts to shape Medicaid policy: *Olmstead v. L. C.* by Zimring and its community integration legacy. J Heal Polit Policy Law. 2016;41(4):585–97. 10.1215/03616878-3620833 27127257

[45] Warshawsky MJ. The second failed attempt at public insurance for long-term services and supports. Health Affairs Forefront [blog on the Internet]. 2022 Feb 3 [cited 2022 Jul 22]. Available from: https://www.healthaffairs.org/do/10.1377/forefront.20220131.939312/

[46] Fox-Grage W. Medicaid: a last resort for people needing long-term services and supports [Internet]. Washington (DC): AARP Public Policy Institute; 2017 Mar [cited 2022 Jul 22]. Available from: https://www.aarp.org/content/dam/aarp/ppi/2017-01/Medicaid-A-Last-Resort-for-People-Needing-Long-Term-Services-and-Supports.pdf

[47] O’Keeffe J, Saucier P, Jackson B, Cooper R, McKenney E, Crisp S, Understanding Medicaid home and community services: a primer [Internet]. Washington (DC): Department of Health and Human Services, Office of the Assistant Secretary for Planning and Evaluation; 2010 [cited 2022 Jul 22]. Available from: https://aspe.hhs.gov/sites/default/files/migrated_legacy_files//43551/primer10.pdf

[48] Thach NT, Wiener JM. An overview of long-term services and supports and Medicaid: final report [Internet]. Washington (DC): Department of Health and Human Services, Office of the Assistant Secretary for Planning and Evaluation, Office of Disability, Aging, and Long-Term Care Policy; 2018 May [cited 2022 Jul 22]. Available from: https://aspe.hhs.gov/sites/default/files/migrated_legacy_files//182846/LTSSMedicaid.pdf

[49] Watts MO, Musumeci M, Chidambaram P. Medicaid home and community-based services enrollment and spending [Internet]. San Francisco (CA): Henry J. Kaiser Family Foundation; 2020 Feb [cited 2022 Jul 22]. Available from: https://files.kff.org/attachment/Issue-Brief-Medicaid-Home-and-Community-Based-Services-Enrollment-and-Spending

[50] Eiken S, Sredl K, Burwell B, Amos A. Medicaid expenditures for long-term services and supports in FY 2016 [Internet]. Baltimore (MD): Medicaid Innovation Accelerator Program; 2018 May [cited 2022 Jul 22]. Available from: http://www.advancingstates.org/sites/nasuad/files/ltssexpenditures2016.pdf

[51] Medicaid and CHIP Payment and Access Commission. State management of home- and community-based services waiver waiting lists [Internet]. Washington (DC): MACPAC; 2020 Aug [cited 2022 Jul 22]. Available from: https://www.macpac.gov/wp-content/uploads/2020/08/State-Management-of-Home-and-Community-Based-Services-Waiver-Waiting-Lists.pdf

[52] Musumeci M, Watts MO, Chidambaram P. Key state policy choices about Medicaid home and community-based services [Internet]. San Francisco (CA): Henry J. Kaiser Family Foundation; 2020 Feb [cited 2022 Jul 22]. Available from: https://files.kff.org/attachment/Issue-Brief-Key-State-Policy-Choices-About-Medicaid-Home-and-Community-Based-Services

[53] Keisler-Starkey K, Bunch LN. Health insurance coverage in the United States: 2020 [Internet]. Washington (DC): Census Bureau; 2021 Sep [cited 2022 Jul 22]. Available from: https://www.census.gov/content/dam/Census/library/publications/2021/demo/p60-274.pdf

[54] Rosenbaum SD. Appendix D. The Americans with Disabilities Act in a health care context, In: Field MJ, Jette AM, editors. The future of disability in America. Washington (DC): National Academies Press; 2007. p. 426–52. 20669428

[55] Cornachoine E, Musumeci MB, Artiga S. Summary of HHS’s final rule on nondiscrimination in health programs and activities [Internet]. Washington (DC): Kaiser Commission on Medicaid and the Uninsured; 2016 Jul [cited 2022 Jul 22]. Available from: https://files.kff.org/attachment/issue-brief-Summary-of-HHSs-Final-Rule-on-Nondiscrimination-in-Health-Programs-and-Activities

[56] Musumeci MB, Kates J, Dawson L, Salganicoff A, Sobel L, Artiga S. The Trump administration’s final rule on Section 1557 non-discrimination regulations under the ACA and current status [Internet]. San Francisco (CA): Henry J. Kaiser Family Foundation; 2020 Sep 18 [cited 2022 Jul 22]. Available from: https://www.kff.org/racial-equity-and-health-policy/issue-brief/the-trump-administrations-final-rule-on-section-1557-non-discrimination-regulations-under-the-aca-and-current-status/

[57] Musumeci MB, Dawson L, Sobel L, Kates J. Recent and anticipated actions to reverse Trump administration Section 1557 non-discrimination rules [Internet]. Washington (DC): Henry J. Kaiser Family Foundation; 2021 Jun 9 [cited 2022 Jul 22]. Available from: https://www.kff.org/racial-equity-and-health-policy/issue-brief/recent-and-anticipated-actions-to-reverse-trump-administration-section-1557-non-discrimination-rules/

[58] Department of Health and Human Services. Patient Protection and Affordable Care Act; HHS notice of benefit and payment parameters for 2023. Fed Regist. 2022;87(88):27208–393.

[59] National Council on Disability. NCD education forum report: UN Convention on the Rights of Persons with Disabilities [Internet]. Washington (DC): NCD; 2013 Dec [cited 2022 Jul 22]. Available from: https://ncd.gov/publications/2013/12122013

